# Autoimmune retinopathy: A Review

**DOI:** 10.1186/s40942-017-0104-9

**Published:** 2018-01-03

**Authors:** Aristófanes Mendonça Canamary, Walter Yukihiko Takahashi, Juliana Maria Ferraz Sallum

**Affiliations:** 1 Federal University of São Paulo, Rua Botucatu 821, Vila Clementino, São Paulo, SP CEP: 04023-062 Brazil; 2 Medical School of University of São Paulo, Avenida São Gualter 99, Alto Pinheiros, São Paulo, SP CEP:05455-000 Brazil

**Keywords:** Autoantibodies, Recoverin, Immunosuppression, Retinal cone photoreceptor cells, Rod cell outer segment

## Abstract

Autoimmune retinopathy (AIR) is a rare and still poorly understood immune-mediated disease that may cause inflammation from circulating autoantibodies against the retina. It may be related to history of autoimmune disease in the patient or in a family member or the presence of neoplastic disease in the individual. The disease may be subdivided into paraneoplastic and non-paraneoplastic AIR. When related to melanoma, it is referred to as MAR, and when related to other cancers, it is called CAR. The exact prevalence of AIR is unknown. It mainly affects older adults. Patients present with bilateral and asymmetric scotomas, photopsias, visual field defects, with rapidly progressive visual loss in late onset. In the initial stage, fundus examination is unremarkable, and in late stages, there is limited retinal epitheliopathy and vascular attenuation, with or without optic disc pallor, associated or not with intraocular inflammation and with no evidence of degenerative retinal disease. A clinical investigation with detailed anamnesis and laboratory tests should be performed to search for an associated neoplasm. Ophthalmologic and complementary examinations such as full-field electroretinogram, optical coherence tomography, visual field and fundus autofluorescence, help the diagnosis. Blood tests to search for autoantibodies should be requested. Management consists of prolonged immunosuppression, which may be combined with antioxidant vitamins. In general, the prognosis is uncertain, so the disease still needs to be better understood. More studies should be performed to improve diagnostic measures and define specific management that could preserve or even restore vision.

## Background

Autoimmune retinopathy (AIR) is a rare inflammatory condition that can lead to blindness, and while it has been studied for several years, it still remains under diagnosed [1]. It had been associated with an immune-mediated component and is related to circulating antiretinal antibodies, which are believed to be responsible for the retinopathy [2, 3], although the precise mechanisms are not entirely understood.

The initial signs and symptoms depend on the type of cell most affected and the antiretinal antibody involved, causing a diverse clinical, anatomical and functional presentation [4].

AIR is characterized by usually bilateral, suddenly progressive, painless visual deterioration [5], scotomas, visual field defect and retinal (photoreceptor) dysfunction. The clinical examination may show some abnormalities such as little or no retinal pigment epitheliopathy, vascular attenuation, optic disc pallor, with minimal or no ocular signs of inflammation [6]. Although these alterations have been described, most of the cases have no ocular findings on fundus examination [7]. History of cancer, autoimmune diseases in the family, presence of circulating autoantibodies against the retina, progressive visual loss, associated with alterations in full-field ERG, with the presence or not of the ophthalmologic findings described above, with no evidence of degenerative eye disease, may lead to a diagnosis of AIR [2, 6]. OCT [8], visual field, and FAF help the diagnosis, and a search for an associated or not neoplastic condition needs to be performed [9, 10]. This retinopathy can be divided in two main forms: presumed non-paraneoplastic AIR (npAIR) and paraneoplastic AIR [11]. The first one is not related to cancer and is probably the most common. The second includes cancer-associated retinopathy (CAR) and melanoma-associated retinopathy (MAR), viteliform maculopathy, and bilateral diffuse uveal melanocytic proliferation. CAR and MAR represent the most common paraneoplastic group (Table 1) [3, 6]. Though less common, paraneoplastic syndromes may affect the optic nerve as primary site, a condition known as paraneoplastic optic neuropathy (PON) [12].

**Table 1:** Types of autoimmune retinopathy

Type of autoimmune retinopathy	Associated conditions
Non-paraneoplastic	Not related to cancer
Paraneoplastic	CAR, MAR, viteliform maculopathy, bilateral diffuse uveal melanocytic proliferation

The presence of more than one circulating autoantibody in AIR leads to an overlap of clinical features and may even be similar to degenerative retinal disorders [6]. In addition, the lack of standardized diagnostic criteria and its rare incidence make diagnosis even more difficult [6]. Appropriate clinical assessment, understanding the pathophysiology, can help achieve a faster diagnosis, so that appropriate management could be started earlier in the course of the disease, thereby increasing the chance of preventing or decreasing visual worsening.

The aim of this study was to better understand this disease to achieve an earlier diagnosis and initiate the appropriate management more quickly, but even so, further research are needed.

## Epidemiology

The exact prevalence of this disease is unknown. It mainly affects older adults. A review of 209 patients with non-paraneoplastic retinopathy determined an average age at diagnosis of 65 years, where women were the most affected [5], although other authors reported a case in a 3-year-old child [7]. Presumed non-paraneoplastic retinopathy is the most prevalent of all forms of AIR. MAR seems to affect more men, due to melanoma being more prevalent in men, and is believed to be increasing in frequency relative to CAR. Even so, CAR is currently still the most common form of paraneoplastic retinopathy [11]. The malignancy most frequently associated with this disorder is small-cell lung cancer, followed by breast and gynecologic (uterine, ovarian and cervical) carcinoma. Other cancer associations include hematological, prostate, colon and lymphomas [5, 13].

## Clinical features

Patients with npAIR typically present with sudden vision loss, scotomas, photopsias, nyctalopia or photoaversion and dyschromatopsia [7]. Signs and symptoms can differ depending on which retinal cells are affected. CAR affects both cones and rods, while MAR characteristically shows rod dysfunction secondary to antibodies toward bipolar cells (Table 2) [3].

**Table 2:** Autoimmune retinopathy and related cell dysfunction

Autoimmune retinopathy	Cell dysfunction
Non-paraneoplastic	Cones or rods or both
*Paraneoplastic*	
CAR	Cones and rods
MAR	Rods, antibodies against bipolar cells

*CAR* cancer-associated retinopathy, *MAR* melanoma-associated retinopathy

Photosensitivity, hemeralopia, loss of color vision, and decreased visual acuity and central vision can be experienced with cone dysfunction. Nyctalopia, prolonged dark adaptation, and peripheral field loss are results of rod malfunction [13]. Photopsia occurs in both cases (Table 3). In early stages, there is good visual acuity, and as time goes by, some patients report transient dimming of vision, which may be mistaken for retinal vascular disease such as Behçet and systemic lupus erythematosus [7].

**Table 3:** Retinal cell dysfunction and associated symptoms

Retinal cell dysfunction	Symptoms
Cones	Photopsia, photosensitivity, hemeralopia, loss of color vision, diminished vision acuity, diminished central vision
Rods	Photopsia, nyctalopia, prolonged dark adaptation, peripheral vision field loss

**Fig. 1:**
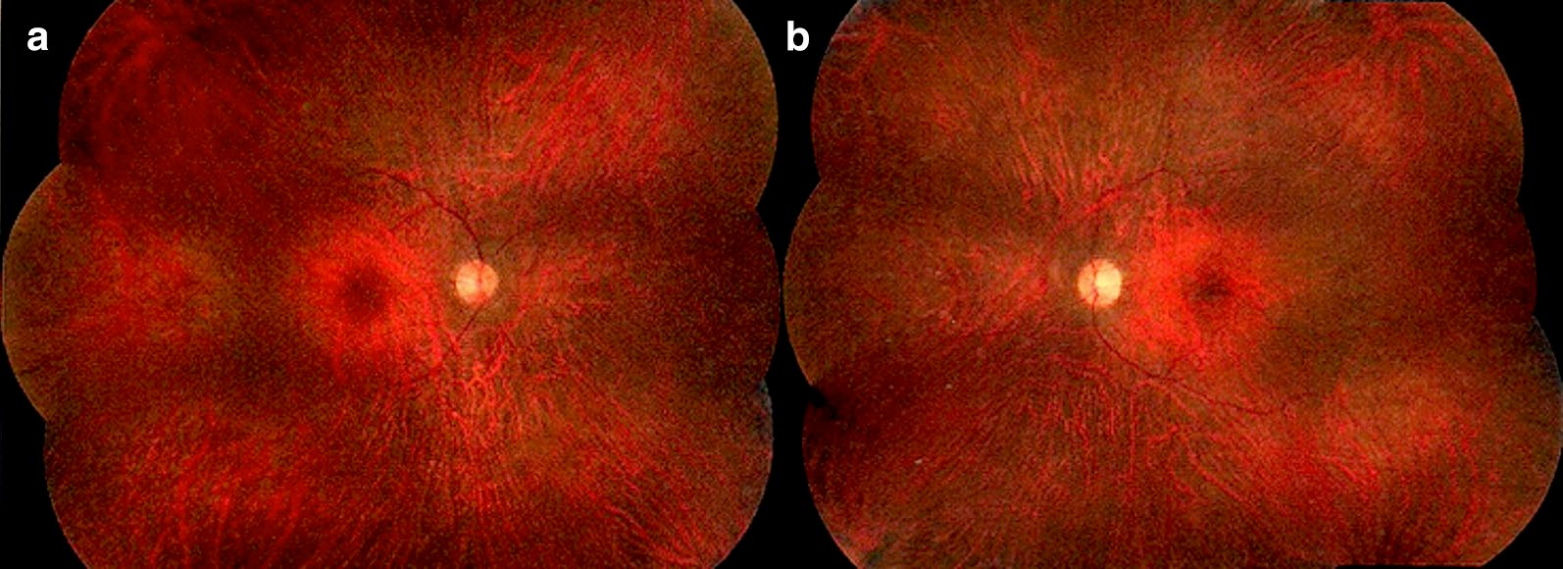
Color fundus. Shows normal appearance of optic disc and macula. Also pigmentary changes in mid-periphery and discrete vascular (arteries) attenuation. A 56-year-old male started to notice 3 years ago, rapid progressive visual field loss and nyctalopia without any other local or systemic symptoms. There were no signs of ocular inflammation and no history of autoimmune diseases or cancer. Blood tests showed some antiretinal antibodies. Fluorescein angiography (FA) showed areas of leakage. Full-field ERG demonstrated non-detectable rod response in both eyes and preserved response of cones in 7% in both eyes. No gene mutation was found in retinal dystrophy panel. Patient noticed some visual improvement with high doses of immunosuppressive drugs

The disease is normally bilateral and it can be asymmetric [6]. Minimal or no signs of intraocular inflammation can be seen [6]. Funduscopic findings at presentation are unremarkable in all forms of AIR [3]. However, changes may occur over time, including vascular attenuation, diffuse retinal atrophy, mottling of the retinal pigment epithelium and occasionally optic disc pallor (Fig. 1) [13].

In cases of PON, the optic disc can appear normal, edematous or atrophic, accompanied by vitreous cells, and retinal vascular leakage, with bilateral subacute, painless and progressive visual loss in a few days [14].

## Diagnosis

At the onset of the disease, the absence of clinical findings makes the diagnosis challenging. However, patients who present with progressive visual loss, with no previous history, with an apparently normal eye fundus, will be highly suspected of AIR [3].

**Fig. 2:**
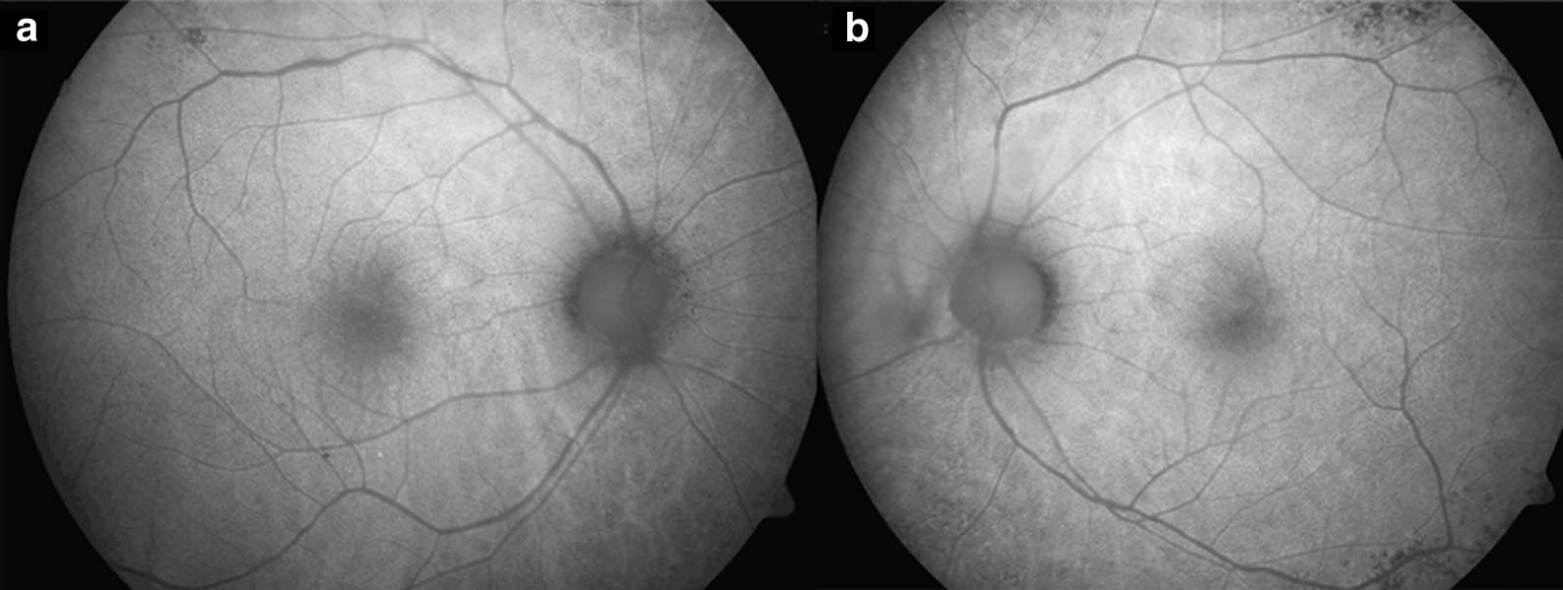
Fundus auto fluorescence. Areas of hypo- and hyperautofluorescence in mid-periphery in both eyes

A thorough assessment of the patient’s visual function should be performed. Complementary examinations such as visual field, color vision tests, FAF (Fig. 2), FA, OCT, and, in some cases, full-field ERG can be helpful [5].

Visual field testing shows constriction and central or paracentral scotomas [7]. There are no pathognomonic electrophysiological findings in AIR. The literature has few data and the small studies and reports of existing cases demonstrate and suggest heterogeneity in the electrophysiological features. Full-field ERG shows abnormalities and specific findings depending on the predominance of cone, rod and other neural elements dysfunction [6, 13]. There are reports with delayed b-wave, reduced amplitudes of both a- and b-waves, reduced b-wave, and an electronegative ERG, in patients with CAR [15]. More homogeneous ERG patterns are seen in patients with MAR, with most of them having markedly reduced b-wave but a normal dark-adapted a-wave (negative ERG appearance) [16]. Full-field ERG was extinguished in one patient and selective b-wave loss in other patients with npAIR [17].

**Fig. 3:**
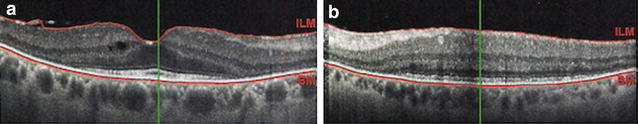
Optical coherence tomography. Images shows loss of ellipsoid zone and photoreceptors in both eyes

FA is performed to exclude other potential causes of vision loss [13]. In cases of vasculitis, leakage occurs. OCT demonstrates a thinning of the inner retinal layers [13]. FAF shows an abnormal autofluorescence pattern, mainly in the form of a hyperautofluorescent ring in the parafoveal region, in which OCT shows loss of the inner segment and outer segment (IS/OS) junction (Fig. 3) and thinning of the outer nuclear layer [7].

As there is no standardization of the diagnostic criteria, the clinical characteristics may vary. The identification of circulating antiretinal antibodies contributes to the diagnosis of AIR [7]. Some healthy individuals may occasionally have these antibodies without clinical evidence of retinopathy [7], and in some cases, antibodies cannot be detected with current techniques such as Western blot, immunohistochemistry or enzyme immunoassay [7]. Sometimes patients with presumed CAR may not have detectable antiretinal antibodies [6], which could be as many as one-third such patients (Table 4) [13].

**Table 4:** Main antibodies against retinal proteins associated with AIR

Retinal proteins	Main antibodies
Antibodies	Anti-recoverin, anti-alpha-enolase, anti-transducin, anti-CAII, anti-arrestin, anti-rhodopsin, anti-Muller glial cells, anti-mitofilin, anti-tintin, anti-COX

*COX* cytochrome c oxidase, *CAII* carbonic anhydrase II

The most sensitive and specific antibody for CAR is against the retinal protein recoverin (23 kDa), which is expressed by numerous tumors [13, 18, 19]. Another autoantibody identified in both CAR and npAIR patients is directed against alpha-enolase (46-kDa) [9].

Antibodies to rod transducin and carbonic anhydrase II (CAII) have been identified in patients with CAR, MAR and npAIR [5].

Some patients with MAR have been found to express a variety of autoantibodies against various proteins including transducin, rhodopsin, arrestin, a 35-kDa protein in Müller glial cells, mitofilin (mitochondrial protein), titin and COX (cytochrome c oxidase, assembly mitochondrial protein) [5].

The antibody that defines PON is collapsin response mediator protein-5 (CRMP-5) [12, 14]. Yu et al. [20], reported CRMP-5 positive 116 cases that were associated with different neurologic symptoms.

Patients with anti-recoverin have a more rapid onset and visual decline compared to anti-alpha-enolase [13].

Usually, the symptoms occur prior to cancer diagnosis, so that vision loss and the identification of autoantibodies in case of CAR precede the diagnosis of systemic malignancy by months to years [13]. Unlike with CAR, patients presenting with MAR have previously diagnosed cutaneous melanoma, where a case series showed a mean latency of 3 and a half years [16]. Visual loss may be the only symptom in PON or it can be associated with neurological dysfunctions such as dizziness, dementia, cerebellar and cognitive findings, and motor and sensory abnormalities [12]. Polyneuropathy, organomegaly, endocrinopathies, monoclonal gammopathies, may be considered in cases that present with neuro-ophthalmological signs and symptoms [14].

In any patient with suspected CAR and without a known malignancy, a full systemic evaluation with a complete medical history and physical examination, chest X-ray, and liver enzymes should be performed, and also more specific and appropriate examinations should be carried out according to the anamnesis [6, 7, 19]. In the case of PON, spinal fluid evaluation should be requested, and it may reveal lymphocytosis or elevated protein [14]. Therefore, a good multidisciplinary communication between the ophthalmologist and clinician is essential for the management of these patients.

## Management

AIR is a systemic disease [6]. The diagnosis should be established for proper management to be instituted. Until now there is no standardized therapy or established management protocol in this disease [3], and the literature shows only a few reports and case series as evidence base. In general, the prognosis for these patients is not good [13], and it appears that the final visual result is associated with the type of circulating antibody. Those in whom AIR is associated with anti-alpha-enolase have a worse prognosis than with anti-recoverin [4].

Once the diagnosis is established, short-term management can be initiated with intravitreal and sub-Tenon’s triamcinolone. This does not treat the cause of the disease, since it is a systemic condition, but some specialists try such drugs to help confirm the diagnosis before starting systemic long-term management with immunosuppression, which presently shows the best results [6].

Immunosuppression therapy usually takes more than a year, with corticosteroids and various immunomodulatory drugs, including azathioprine, intravenous immunoglobulin, mycophenolate mofetil, cyclosporine, infliximab, and combinations thereof have been used. Modest visual recovery can occur in some cases, but disease stabilization is the most common outcome [13].

Plasmapheresis can also be used as a management, but it is believed to be more effective when used before visual loss [5]. This seems to decrease the circulating antibodies and thus the damage to photoreceptors [13].

Combined therapy with immunomodulators was used in a study with 24 patients, and in most of them, it was possible to demonstrate a favorable response in both visual acuity and visual field [21].

**Fig. 4:**
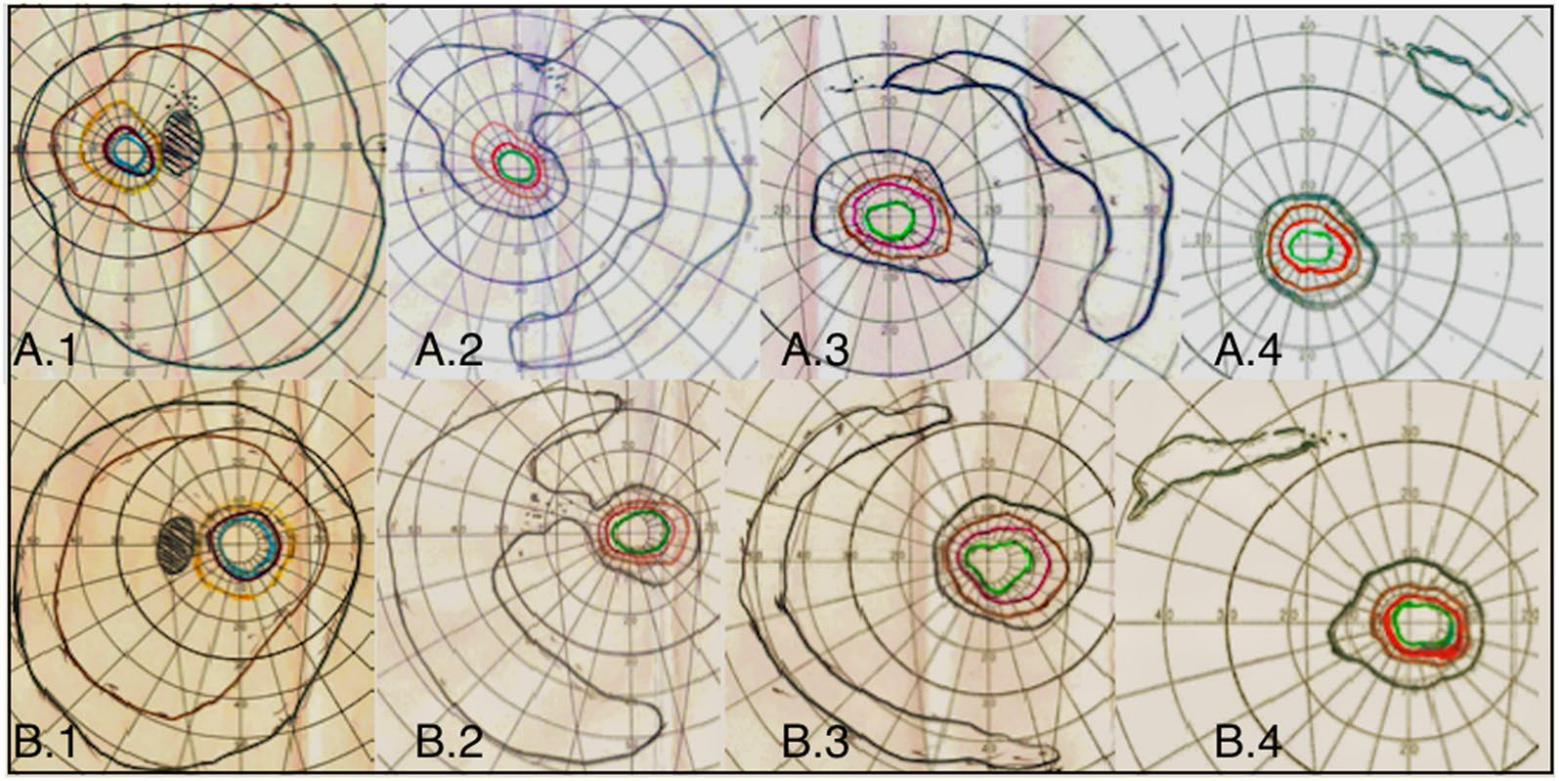
Visual field. Progression of visual field loss at the first examination (**A.1**, **B.1**), after 6 (**A.2**, **B.2**), 8 (**A.3**, **A.3**) and 22 (**A.4**, **B.4**) months follow-up in both eyes in a patient with high suspicion of autoimmune retinopathy

Examinations such as visual field (Fig. 4) and full-field ERG can be repeated every 3–6 months and serve to monitor management response [3]. OCT can also be used to evaluate cystoid macular edema [6].

Management of the primary tumor with surgery, chemotherapy, and radiotherapy does not appear to alter the visual prognosis [13].

Combined with immunosuppression therapy, supplementation with antioxidant vitamins such as beta-carotene (for non-smokers), lutein, vitamin C, and vitamin E (for non-cardiac patients) seems to be important against retinal degeneration [6].

A multidisciplinary team is important for the appropriate management and control of the disease. An oncologist can contribute to the investigation of possible neoplasia, in the cases of paraneoplastic AIR, and in addition, a rheumatologist is more accustomed to the use of immunosuppressive drugs and therefore more familiar with their doses and side effects.

## Conclusion

Currently, there are no standard clinical and laboratory guidelines for this entity. The disease needs to be better understood to obtain a global consensus on diagnosis, management and prognosis. Further research is needed on the actual benefit of decreasing circulating antibodies for visual improvement. At present, the response to management is variable. In most cases, visual prognosis is uncertain, and an improvement in visual field and visual acuity is observed in some patients, while there is still no specific therapy. Even experienced ophthalmologists may have difficulty diagnosing this disease because of its rarity and lack of understanding it.

Accordingly, we need to improve diagnostic measures and their efficacy as well, so the management and appropriate ocular and systemic therapy can be initiated and vision possibly preserved or even restored.

## Abbreviations

AIR: Autoimmune retinopathy
CAII: Carbonic anhydrase II
CAR: Cancer-associated retinopathy
COX: Cytochrome c oxidase
CRMP-5: Collapsin response mediator protein 5
ERG: Electroretinogram
FA: Fluorescein angiography
FAF: Fundus autofluorescence
IS: Inner segment
MAR: Melanoma-associated retinopathy
npAIR: Non-paraneoplastic autoimmune retinopathy
OCT: Ocular coherence tomography
OS: Outer segment
PON: Paraneoplastic optic neuropathy
